# Effects of exercise modalities on decreased blood pressure in patients with hypertension

**DOI:** 10.3389/fphys.2022.993258

**Published:** 2022-10-14

**Authors:** Thiago Rozales Ramis, Franccesco Pinto Boeno, Rodrigo Leal-Menezes, Samuel Vargas Munhoz, Juliano Boufleur Farinha, Jerri Luiz Ribeiro, Alvaro Reischak-Oliveira

**Affiliations:** ^1^ Exercise Research Laboratory, School of Physical Education, Physiotherapy and Dance, Universidade Federal do Rio Grande do Sul, Porto Alegre, Brazil; ^2^ Department of Applied Physiology and Kinesiology, University of Florida, Gainesville, FL, United States

**Keywords:** hemodynamics, nitric oxide, endothelin-1, physical activity, cross-sectional studies, ambulatory blood pressure monitoring

## Abstract

This study aimed to evaluate the acute effects of aerobic and resistance exercises on blood pressure and endothelial blood markers. We also correlated post-exercise blood pressure response with baseline cardiovascular parameters in middle-aged patients with hypertension. This cross-sectional study randomized 54 volunteers into the aerobic exercise group (AG, *n* = 27; 45.6 ± 7.7 years) or dynamic resistance exercise group (RG, *n* = 27; 45.8 ± 8.4 years). Blood marker evaluation, cardiopulmonary exercise tests, resting blood pressure monitoring, ambulatory blood pressure monitoring (ABPM), flow-mediated dilatation monitoring, and body composition evaluation were carried out. Exercise sessions were performed to evaluate post-exercise hypotension (PEH) and endothelial marker responses, in addition to post-exercise ABPM (ABPMex). This study is an arm of the study which was approved by the local ethics committee (No. 69373217.3.0000.5347) in accordance with the Helsinki Declaration and was registered at ClinicalTrials.gov (NCT03282942). The AG performed walking/running at 60% of the reserve heart rate, while the RG performed 10 exercises with two sets of 15–20 repetitions. The mean 24 h ABPM and ABPMex values showed no significant statistical differences. Systolic and diastolic blood pressure hypotension after aerobic and dynamic resistance were −10.59 ± 5.24/−6.15 ± 6.41 mmHg and −5.56 ± 7.61/−6.20 ± 8.25 mmHg, respectively. For an up-to-7 h assessment of resting pressure, there was a positive effect in the aerobic group. The concentrations of nitrites/nitrates (NOx) and endothelin-1 (ET-1) did not change during hypotension. Moreover, PEH and ABPMex were significantly correlated with baseline health variables. Thus, when middle-aged patients with hypertension perform aerobic or resistance exercise, the NOx/ET-1 pathway does not provide the best explanation for PEH. Finally, we found associations between baseline cardiovascular variables and endothelial vasoconstrictors with PEH.

## Introduction

Physical exercise is a non-pharmacological strategy that can, directly and indirectly, assist in the treatment of hypertension. Aerobic exercise promotes post-exercise hypotension (PEH), even at low intensity and short duration (Kenney and Seals, 1993; Pascatello et al., 2016). However, the magnitude of this phenomenon is inconsistent among studies following a dynamic resistance exercise session. Recently, it was demonstrated that PEH occurs after one or three sets of resistance exercises in women with hypertension (De Freitas Brito et al., 2019). Additionally, the magnitude of PEH seems to be associated with the chronic effects of training and consequent resting blood pressure values (Hecksteden et al., 2013; Brito et al., 2018a). Thus, investigating the PEH phenomenon is relevant, as this effect is important for chronic adaptation.

In the last decade, PEH has been investigated using different aerobic exercise models in middle-aged patients with hypertension (Augeri et al., 2009; Eicher et al., 2010; New et al., 2013; Simoes et al., 2013; De Brito et al., 2015; Cunha et al., 2016; Pescatello et al., 2016; Morales-Palomo et al., 2017; Pescatello et al., 2017; Brito et al., 2018b; Da Silva et al., 2018; Fonseca et al., 2018; Joubert et al., 2018; Neto et al., 2018; Ramirez-Jimenez et al., 2018; Cilhoroz et al., 2019; Pimenta et al., 2019). However, only one study has used heart rate reserve to control exercise intensity (Ciolac et al., 2009). This is important because reserve heart rate is recommended for exercise intensity control in patients with hypertension (SBC, 2010a). However, only nine studies looking at the PEH following resistance exercises in middle-aged patients with hypertension were found (Tibana et al., 2013; De Freitas Brito et al., 2015; Queiroz et al., 2015; Figueiredo et al., 2016; La Scala Teixeira et al., 2017; Queiroz et al., 2017; De Freitas Brito et al., 2019; Zafeiridis et al., 2019; Boeno et al., 2021). Regarding exercise integrity control, the moderate fatigue method has been suggested as an alternative to avoid concentric failure and consequent blood pressure peaks during resistance exercise sessions (Queiroz et al., 2015; La Scala Teixeira et al., 2017; Queiroz et al., 2017; De Freitas Brito et al., 2019). Additionally, PEH is usually measured for a short period of time, and studies showing ambulatory blood pressure monitoring (ABPM) responses to different exercise modalities are needed. (Cardoso et al., 2010; Carpio-Rivera et al., 2016; Casonatto et al., 2016; Coelho-Júnior et al., 2022).

The physiological mechanisms by which exercise promotes PEH suggest decreased cardiac output (CO) and peripheral vascular resistance (PVR). Patients with hypertension present with an exacerbated sympathetic tone, while baroreflex control and endothelial function are impaired. Consequently, the PVR may be affected, and a decrease in CO can be facilitated (Brito et al., 2014). However, the literature presents two important mechanisms for lowering blood pressure: 1) immediate post-exercise hyperemia and 2) sustained post-exercise vasodilation (Halliwill et al., 2013; Romero et al., 2017). Therefore, the balance between the vasodilators and vasoconstrictors is important. This balance may be associated with PVR to explain the physiological mechanisms of PEH.

Endothelium-derived substances, such as nitrites/nitrates (NOx) and endothelin-1 (ET-1), are important markers of vascular tone homeostasis (Shah, 2007; Green et al., 2011). However, in the context of hypertension, endothelial tissue may present with functional impairments, such as reduced bioavailability of nitric oxide (NO) and increased ET-1 concentrations, compromising the endothelium’s ability to respond to hemodynamic stimuli (Green et al., 2011; Konukoglu and Uzun, 2017). In this way, prehypertensive subjects who performed aerobic exercise for 30 min presented with PEH; however, NOx levels remained unchanged until 2 h after session cessation (New et al., 2013). In contrast, a recent study by our group showed increased ET-1 levels after high-intensity dynamic resistance exercises in sedentary men. Individuals who performed moderate-intensity exercise demonstrated increased flow-mediated dilation (FMD) at 30 min and NOx levels immediately after the workout session (Boeno et al., 2019).

To the best of our knowledge, no studies have investigated the balance between NO and ET-1 derived from the endothelium during PEH in middle-aged subjects with hypertension under antihypertensive drug treatment. Therefore, the present study aimed to evaluate the effect of a single session of aerobic and dynamic resistance exercise on clinical, ABPM, and endothelial marker levels in middle-aged patients with hypertension. We also sought to correlate post-exercise blood pressure responses with the baseline parameters of lipid profile, endothelial function, and endothelial markers.

## Materials and methods

### Study population

**FIGURE 1:**
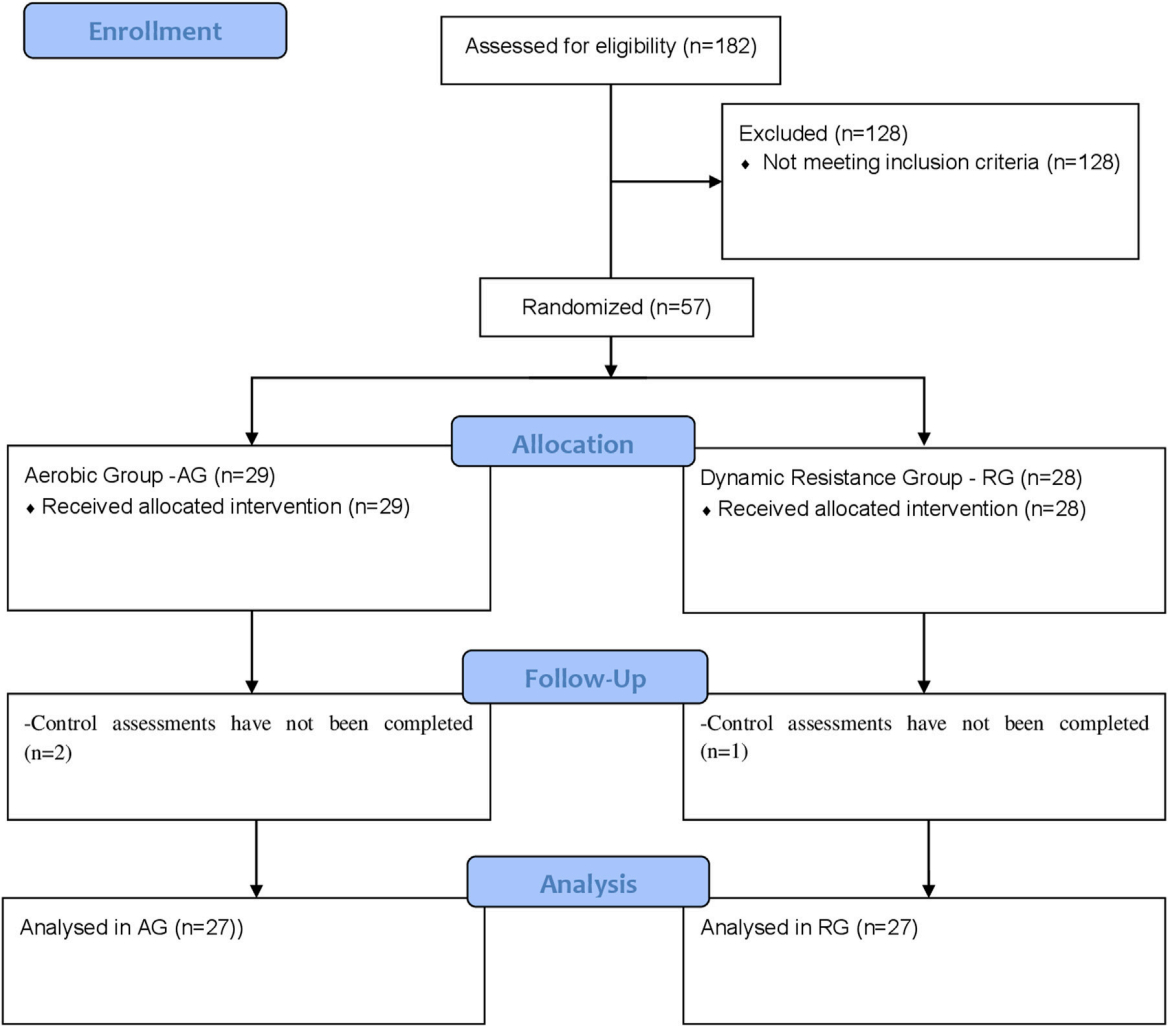
Flowchart of the recruitment, allocation, follow-up, and analysis of participants’ data processes.

Participants were recruited from the community using flyers and advertisements on the radio/internet, in newspapers, and in magazines. A total of 182 individuals met the preliminary study criteria and were invited to complete laboratory screening. They were fully informed about the study procedures and potential risks/benefits, and they gave written informed consent. An interview was conducted to confirm the eligibility criteria. This included a comprehensive medical history and information regarding medication use and exercise participation (Figure 1).

Men and women with hypertension aged 30–59 years, who were receiving at least one antihypertensive drug, were enrolled in the study. Patients were excluded based on the following criteria: 1) body mass index ≥40 kg/m^2^; 2) participation in regular exercise training of any type in the previous 6 months; 3) symptomatic peripheral arterial occlusive disease; 4) aortic insufficiency or stenosis greater than stage I; 5) hypertrophic obstructive cardiomyopathy; 6) congestive heart failure (NYHA II); 7) uncontrolled cardiac arrhythmia with hemodynamic relevance; 8) change in antihypertensive drugs in the preceding 4 weeks and during the study, 9) unstable coronary artery disease, or 10) the use of tobacco products.

Participants’ characteristics are presented in Table 1. Fifty-four participants (24 women and 30 men) were randomized (https://www.randomizer.org/) into two experimental groups: the aerobic group (AG) and the dynamic resistance group (RG). In addition, the individuals in the experimental groups were their own controls, called aerobic control (AC) and resistance control (RC), respectively. The examiner was blinded to the group assignment of the patients and did not participate in the intervention. This study is an arm of the study which was approved by the local ethics committee (Nº:69373217.3.0000.5347) in accordance with the Helsinki Declaration and was registered at ClinicalTrials.gov (NCT03282942).

**TABLE 1:** Characterization of the middle-aged subjects with the hypertension sample submitted to an aerobic or strength exercise session.

Characteristics of subjects	AG (*n* = 27)	RG (*n* = 27)	*p* value
Age (years)	45.6 ± 7.7	45.8 ± 8.4	0.89
Height (cm)	168.6 ± 9.4	167.1 ± 11.2	0.57
Women, *n* (%)	13 (48.1)	11 (40.7)	—
Men, *n* (%)	14 (51.9)	16 (59.3)	—
ARA, *n* (%)	7 (25.9)	9 (33.3)	—
ECA, *n* (%)	6 (22.2)	3 (11.1)	—
DIU, *n* (%)	1 (3.7)	3 (11.1)	—
ARA + DIU, *n* (%)	6 (22.2)	4 (14.8)	—
ARA + BB, *n* (%)	4 (14.8)	3 (11.1)	—
ARA + DIU + CCB, *n* (%)	0 (0)	1 (3.7)	—
ARA + DIU + BB, *n* (%)	0 (0)	1 (3.7)	—
ECA + DIU, *n* (%)	1 (3.7)	0 (0)	—
ECA + BB, *n* (%)	0 (0)	2 (7.4)	—
ECA + DIU + BB, *n* (%)	1 (3.7)	1 (3.7)	—
DIU + CCB, *n* (%)	1 (3.7)	0 (0)	—

The therapeutic classes were grouped at absolute and relative frequency. Therapeutic class: ARA, angiotensin receptor antagonist; ECE, angiotensin-converting enzyme; DIU, diuretic; BB, beta blocker; CCB, calcium channel blocker.

### Experimental design

This is a cross-sectional study. The primary outcome was post-exercise blood pressure, and the secondary outcome was the changes in the vasoactive substances of the endothelium after exercise. Individuals who met the study criteria (*n* = 57) were randomized into blocks of three according to peak oxygen consumption (VO_2peak_), sex, and body mass index (BMI). Follow-up data were accessed prior to the experimental sessions: venous blood samples, resting blood pressure (72 h without strenuous activities), cardiopulmonary exercise test (CPET), FMD, and body composition. Subsequently, the subjects randomly performed a control or exercise session. Finally, an exercise session corresponding to that of the experimental group was conducted. In both groups, the subjects’ blood samples were collected following an exercise session to evaluate endothelial vasoactive substances. Blood pressure and heart rate were measured following exercise before blood collection. Finally, the participants performed ABPM after the exercise session (ABPMex). For the control session, the subjects performed only ABPM.

Female participants were evaluated between days 1 and 7 of their menstrual cycles to control for the potential influence of hormonal fluctuations during the menstrual cycle (Farinha et al., 2018). Data analysis of all the measures was performed by investigators blinded to the allocation of the participants, and the CPET was analyzed by two independent blinded researchers.

### Exercise protocols

The participants were familiarized with the equipment and exercise protocols for 1 week and then performed their respective experimental conditions as described below.

The AG walked on a treadmill for 5 min to warm-up and walked/ran for 45 min at 60% of the heart rate reserve_._ Heart rate was monitored during the AG session using telemetry (Polar Electro Oy, Kempele, Finland) (2010b, Thompson et al., 2013; Pescatello et al., 2004). To perform the heart rate calculation, the cardiopulmonary exercise test parameters were used.

The RG consisted of two sets (passive rest for 120 s) of 15–20 submaximal repetitions of bench press, leg press, lat pulldown, leg extension, shoulder press, leg curl, biceps curl, plantar flexion, and triceps extension exercises. To avoid pressure peaks during the session, the participants performed the maximum weight movement with a good technique (without concentric failure). In addition, two sets of 15 repetitions of abdominal exercises were performed (2010b, Thompson et al., 2013; Pescatello et al., 2004).

We ensured that the training volumes of both the groups were similar. The interventions were equalized over time (approximately 50 min).

### Blood pressure assessment

The individuals randomly performed a control or exercise session. ABPM was performed in the control session and ABPMex after the experimental (AG or RG) or control (AC or RC) sessions. The evaluations were conducted at an interval of at least 1 week.

Blood pressure was measured every 15 min during the day and every 30 min at night for 24 h using a noninvasive automatic ABPM system (Meditech KFT Ulloiút 200, H-1191 Budapest, Hungary). After this period, the individuals visited the laboratory again for the removal of the equipment, and the recorded data were analyzed using HYPERView 7.0.0 software (MICROMED Biotechnology, Brazil) (O’brien et al., 2013). Nocturnal fall was calculated as [(mean daytime-mean nighttime)/mean daytime] × 100. From an hourly analysis, we used the longest time interval for all participants to provide blood pressure data after the exercise and control sessions.

In addition, office blood pressure and resting heart rate were measured using a validated sphygmomanometer (Omron, HEM-907, Japan), and data were obtained as recommended by the Brazilian Guidelines (SBC, 2010b; SBC2, 2011). Additionally, blood pressure was assessed immediately after the exercise sessions. The participants remained in an air-conditioned and quiet room in a sitting position for 30 min. Measurements were performed immediately after the end of the exercise and after 15 and 30 min using an oscillometric monitor (Omron, HEM-907, Japan) according to the manufacturer’s instructions.

Individuals were familiarized with office blood pressure measurements. In addition, to minimize possible bias in the measurements, it was performed during all the visits. Regarding ambulatory measurements, all subjects performed a control session of ABPM. Finally, ABPM was performed on the same day of the week and time to minimize bias in relation to the routine (De Brito et al., 2019).

### Body composition

The anthropometric evaluation was based on anatomical site markings and the measuring skinfolds technique, following the standards of the International Society for the Advancement of Kinanthropometry (ISAK); therefore, body composition was calculated using a 5-component method (Marfell-Jones et al., 2006).

The standing height, body mass, and waist circumference were measured using the standard anthropometric procedures. For muscle thickness measurements, ultrasound images of the right vastus lateralis (VL), rectus femoris (RF), vastus intermedius (VI), vastus medialis (VM), biceps brachii, and brachialis muscles were obtained, as we previously described (Ramis et al., 2018), using an ultrasound system equipped with a 9.0 MHz linear-array transducer (LOGIQ-E, GE Medical Systems, Milwaukee, United States). Briefly, transverse images were acquired by an experienced investigator with the ultrasound transducer placed perpendicular to the surface of the thigh and arm, while care was taken to avoid compression of the dermal surface. Three images were acquired and exported to a personal computer for analysis by the same investigator using ImageJ software version 1.42 (Maryland City, United States) (Ramis et al., 2018).

### Cardiopulmonary fitness assessment

After performing the body composition evaluation, the CPET assessment was scheduled for another day. VO_2peak_ and maximal heart rates were determined using an incremental exercise test on a treadmill (Inbramed, Porto Alegre, Brazil). The test consisted of a 5 min warm-up starting at 3 km/h and increasing by 0.5 km/h each min to 5 km/h, followed by increases in incline by 2% and speed by 1 km/h every min until volitional exhaustion. Ventilatory parameters were measured continuously breath-by-breath using an open-circuit spirometry system (Quark CPET, Cosmed, Italy). Heart rate was measured continuously using telemetry (Polar Electro Oy, Kempele, Finland) (Rodrigues-Krause et al., 2018).

### Endothelial function

Brachial artery FMD was measured according to established guidelines (Thijssen et al., 2011), in a quiet, temperature-controlled (22°C) room, following abstinence from alcohol and caffeine intake for at least 12 h. An experienced investigator imaged the brachial artery using a high-resolution ultrasound system equipped with a 7–12 MHz linear-array transducer (LOGIQ-E, GE Medical Systems, Milwaukee, United States).

The participant rested in the supine position with the non-dominant arm extended and abducted at approximately 90°, while the brachial artery was imaged 5–10 cm above the antecubital fossa in the longitudinal plane using duplex mode. Once an optimal image was acquired, the position was maintained for the entire test, and images were recorded at the baseline and after 5 min of ischemia. A blood pressure cuff was placed around the largest part of the forearm and inflated to 250 mmHg for 5 min. Brachial artery B-mode images and Doppler velocity waveforms were obtained continuously for 30 s before and 3 min after the cuff release using an angle of insonation of <60°. Brachial artery images were analyzed by a blinded investigator using FloWave.US (Coolbaugh et al., 2016). Arterial diameters were measured as the distance (mm) between the intima and lumen interfaces of the near and far walls. The blood flow was calculated as follows:

$$BF\;\left(ml/\min\right)=blood\;velocity∙\pi ∙{\left(\frac{vessel\;diameter}{2}\right)}^{2}∙60.$$ (1)

FMD was calculated as follows:

$$FMD\;\left(\%\right)=\frac{\left[\left(peak\;diameter-baseline\;diameter\right)\right]*100}{baseline\;diameter}.$$ (2)

### Blood collection and analyses

Venous blood samples were drawn into 4 ml EDTA anticoagulant tubes. After centrifugation, plasma aliquots were frozen at −80°C for further analysis. After at least 10 h of fasting (basal analyses), blood was drawn to determine the ET-1, prostacyclin (PGI2), thromboxane (TXA2), NOx, glucose, high-density lipoprotein (HDL), low-density lipoprotein (LDL), total cholesterol, and triglyceride levels. Regarding the exercise sessions, blood draws were performed before (pre-exercise), immediately after exercise (Sponton et al., 2014), and 30 min after the session for the measurement of ET-1 and NOx concentrations. ET-1 was determined using ELISA, according to the manufacturer’s instructions (BosterBio, Pleasanton, CA, United States). Plasma levels of PGI2 and TXA2 were detected based on the presence of their stable metabolites, 6-keto-PGF1 and TXB2, respectively, using commercially available ELISA kits (Cayman, Ann Arbor, United States), according to the manufacturer’s protocol. Nitrite and nitrate (NO_x)_ levels were measured using a colorimetric method with commercially available kits (Cayman, Ann Arbor, MI, United States). The absorbance of vascular parameters was measured using a microplate reader (Multiskan Go, Thermo Scientific, Waltham, United States). The glucose, total cholesterol, HDL, and triglyceride levels were measured using an automated analyzer (Cobas C111; Roche Diagnostics, Basel, Switzerland), while the low-density lipoprotein (LDL) levels were estimated using the Friedewald equation (Friedewald et al., 1972).

### Statistical analysis

The sample size was calculated using WinPEPI version 11.65. To perform this calculation, we used preliminary data from our pilot study (unpublished data). ABPM was the main outcome, and the standard deviation of systolic blood pressure (SBP) at 24 h was 6.7 mmHg. To provide 80% power to detect a difference of 2 mmHg between the two groups, a total of 60 individuals would be required to sufficiently power the study.

Data were structured and analyzed using the IBM SPSS statistical package (Statistical Package for Social Sciences, version 22.0, IBM, United States). The Shapiro–Wilk test was performed to verify data normality, while the analysis of the homoscedasticity of variances and sphericity was determined using the Levene’s and Mauchly’s tests, respectively. When comparing the two experimental groups, Student’s t-test was used for independent data on variables with parametric distribution, and the Mann–Whitney U test for variables with non-parametric distribution. The area under the curve (AUC) of mean blood pressure (MBP) was calculated using the trapezoidal method by subtracting the basal levels of MBP from each evaluation point. The comparison of the data with more than one moment was performed using the model of generalized estimation equations (GEE), adopting the factor group (two stratifications) and time (two, three, four, or seven stratifications). When necessary, the Bonferroni *post-hoc* test was used to locate the differences. All results are expressed as mean and standard deviation or standard error, and the significance level was set at 5%. The variables of interest were correlated with PEH and ABPMex through the Pearson or Spearman test and were classified as small (0.3–0.5), moderate (0.5–0.7), or large (≥0.7) correlation (Hinkle et al., 2003).

## Results

Table 1 presents the characteristics of the sample and the use of antihypertensives. There were no differences in body composition, resting blood pressure, muscle thickness, and VO_2peak_ (Table 2). Additionally, no differences were found in endothelium-derived substances, lipid profiles, or FMD (Table 3). Table 4 presents the results for the mean 24-h ABPM and ABPMex values. No differences in ABPM or ABPMex were found between the groups. Regarding daytime, the average heart rate (HR) in the AG was higher in ABPMex than in ABPM. Higher percentages of falls in nighttime HR and rate pressure product were observed after ABPMex when compared to ABPM. In the AG, average drop in HR and rate pressure product after ABPM were 8.58 ± 8.19% and 19.62 ± 10.81% to 15.15 ± 7.15% (*p* < 0.0001) and 25.45 ± 10.05% (*p* = 0.019) in the ABPMex, respectively. On RG, average drop in HR and rate pressure product after ABPM were 10.44 ± 7.36% and 21.14 ± 10.94% to 14.47 ± 9.09% (*p* = 0.042) and 24.83 ± 9.45% (*p* = 0.099) in the ABPMex, respectively (Table 4).

**TABLE 2:** Evaluation of body composition, blood pressure, muscle thickness, and maximum oxygen consumption in middle-aged subjects with hypertension.

Variables	AG (*n* = 27)	RG (*n* = 27)	*p* value
BM (kg)	96.40 ± 14.58	92.83 ± 17.32	0.46
BMI (kg/m^2^)	33.85 ± 4.01	33.09 ± 4.25	0.50
AM (kg)	31.49 ± 6.29	30.88 ± 5.20	0.69
MM(kg)	40.59 ± 7.72	38.73 ± 9.97	0.44
Waist circumference(cm)	100.46 ± 10.27	98.90 ± 11.11	0.59
Hip circumference(cm)	113.10 ± 9.40	110.39 ± 8.82	0.27
SBP rest (mmHg)	124.57 ± 10.10	125.29 ± 10.93	0.80
DBP rest (mmHg)	77.72 ± 10.88	78.18 ± 10.35	0.87
HR rest (BPM)	71.38 ± 9.11	71.97 ± 12.64	0.84
VO_2_ (ml kg^−1^ min^−1^)	26.62 ± 6.03	27.12 ± 6.09	0.76
Biceps (mm)	23.95 ± 5.38	22.15 ± 5.91	0.24
QUAD (mm)	81.07 ± 16.80	76.28 ± 13.81	0.22

BM, body mass; BMI, body mass index; AM, adipose mass; MM, muscle mass; SBP, systolic blood pressure; DBP, diastolic blood pressure; HR, heart rate; QUAD., quadriceps sum; VO_2_, peak oxygen consumption; AG, aerobic exercise group; RG, dynamic resistance exercise group.

**TABLE 3:** Biochemical variables related to vascular homeostasis, lipidic profile, and flow-mediated dilatation in middle-aged subjects with hypertension.

Variables	AG (*n* = 27)	RG (*n* = 27)	*p* value
NOx (μM)	10.46 ± 4.54	10.58 ± 4.15	0.92
ET-1 (pg/ml)	6.04 ± 1.06	5.97 ± 0.92	0.76
Prostacyclin (pg/ml)	6.38 ± 3.41	4.92 ± 2.06	0.11
Thromboxanes (pg/ml)	139.14 ± 100.77	153.87 ± 144.57	0.74
Glucose (mg/dl)	96.69 ± 9.83	96.58 ± 9.20	0.96
Triglycerides (mg/dl)	125.66 ± 45.19	124.89 ± 42.26	0.94
Cholesterol (mg/dl)	198.16 ± 37.31	205.64 ± 41.35	0.48
HDL (mg/dl)	38.34 ± 11.66	38.26 ± 11.95	0.97
LDL (mg/dl)	132.80 ± 38.69	140.51 ± 41.43	0.48
Basal Diameter (mm)	37.22 ± 5.95	37.71 ± 6.55	0.77
Peak diameter time (sec)	85.44 ± 15.84	82.33 ± 17.89	0.50
FMD (%)	7.45 ± 3.35	7.26 ± 3.25	0.85

NO_X:_ nitrite and nitrates; ET-1, endothelin-1; HDL, high-density lipoprotein; LDL, low-density lipoprotein; FMD, flow-mediated dilation; AG, aerobic exercise group; RG, dynamic resistance exercise group.

**TABLE 4:** Ambulatory blood pressure at rest and post-exercise in middle-aged patients with hypertension.

Variables	AG (*n* = 27)	RG (*n* = 27)	P group	AG (*n* = 27)	RG (*n* = 27)	P group	Time AG	Time RG
24 h mean (mmHg)	ABPM			ABPM_ex_				
SBP (mmHg)	119.00 ± 8.66	119.95 ± 8.87		117.75 ± 6.00	120.26 ± 7.72			
DBP	70.71 ± 7.96	72.80 ± 8.20		71.41 ± 6.36	73.51 ± 7.60			
MBP	86.82 ± 7.42	88.57 ± 7.82		86.86 ± 5.42	89.09 ± 7.28			
HR	74.34 ± 9.07	72.98 ± 10.31		74.95 ± 8.54	74.75 ± 9.34			
RPP	8896.82 ± 1327.05	8827.79 ± 1545.93		8877.68 ± 1051.37	9072.97 ± 1437.37			
Daytime (mmHg)								
SBP	124.41 ± 9.45	125.56 ± 8.79		123.05 ± 5.57	125.40 ± 7.74			
DBP	74.86 ± 8.98	77.15 ± 8.78		75.36 ± 6.48	77.42 ± 8.11			
MBP	91.40 ± 8.41	93.36 ± 8.18		91.26 ± 5.16	93.41 ± 7.62			
HR	76.66 ± 9.07*	75.94 ± 10.84		79.12 ± 9.26*	78.76 ± 10.23		*p* = 0.05	*p* = 0.06
RPP	9548.15 ± 1288.75	9566.83 ± 1585.39		9739.89 ± 1162.75	9916.05 ± 1551.88			
Nighttime (mmHg)								
SBP	109.17 ± 11.15	110.42 ± 11.64		107.74 ± 8.95	110.17 ± 10.53			
DBP	63.09 ± 8.22	65.35 ± 8.91		63.80 ± 7.47	65.79 ± 9.25			
MBP	78.45 ± 8.47	80.38 ± 9.27		78.45 ± 7.34	80.58 ± 9.24			
HR	70.03 ± 10.20	67.83 ± 10.44		66.93 ± 8.44	67.03 ± 9.34			
RPP	7690.77 ± 1596.06	7541.45 ± 1656.29		7235.76 ± 1184.71	7433.06 ± 1405.53			
Nocturnal fall (%)								
SBP	12.06 ± 8.42	11.97 ± 8.19		12.44 ± 5.81	12.09 ± 7.01			
DBP	15.28 ± 9.60	14.95 ± 9.97		15.25 ± 7.56	14.81 ± 9.72			
MBP	13.86 ± 8.67	13.71 ± 8.84		14.02 ± 6.45	13.61 ± 8.17			
HR	8.58 ± 8.19*	10.44 ± 7.36**	—	15.15 ± 7.15*	14.47 ± 9.09**		*p* < 0.0001	*p* = 0.042
RPP	19.62 ± 10.81*	21.14 ± 10.94		25.45 ± 10.05*	24.83 ± 9.45		*p* = 0.019	*p* = 0.099

SBP, systolic blood pressure; DBP, diastolic blood pressure; MBP, mean arterial pressure; HR, heart rate; RPP, rate pressure product; AG, aerobic exercise group; RG, dynamic resistance exercise group. *In AG time, the statistical differences (*p* < 0.05). **In RG time, the statistical differences (*p* < 0.05).

**FIGURE 2:**
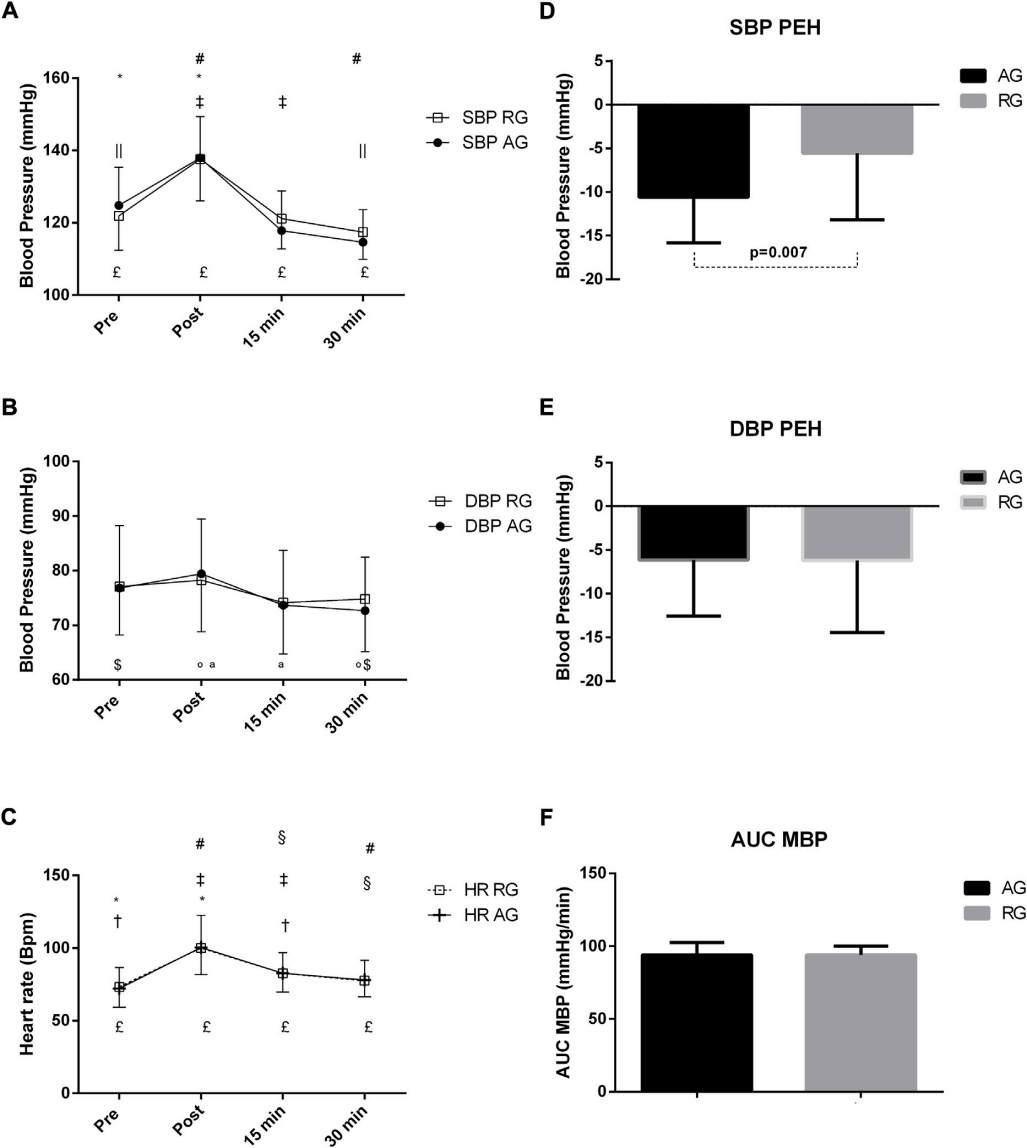
**(A–F)** Responses of systolic blood pressure **(A)**, diastolic **(B)**, heart rate **(C)**, systolic blood pressure hypotension **(D)**, diastolic **(E)**, and area under the curve (AUC) of mean arterial pressure (MBP) **(F)**, after an exercise session in middle-aged patients with hypertension. Subjects were classified into the aerobic exercise group (AG) or dynamic resistance exercise group (RG). In GA, the statistical differences (*p* < 0.05) between moments of acute exercise are represented with symbols in the lower portion of the Figure, **, PRE–POST; ‡‡, PRE—15 min; $, PRE—30 min; ^a^, POST—15 min; °, POST—30 min; &, 15 min—30 min. The symbol £ means that all moments had statistical differences between them. In RG, the statistical differences between moments of the acute exercise with symbols in the upper part of the Figure, *, PRE–POST; †, PRE—15 min; ||, PRE—30 min; ‡, POST—15 min; #, POST—30 min; §, 15 min—30 min.

Figure 2 demonstrates that differences between moments occurred in both groups in terms of SBP. In the AG, differences were found among all moments. In the RG, the pre-exercise value was different from the post and 30 min values, whereas the post-exercise value led to differences when compared to the 15 and 30 min values after exercise. The DBP responses after an aerobic exercise session showed some significant changes, but no changes were found in response to strength exercise. In the AG, there was a DBP decrease between pre and 30 min values (*p* = 0.023), while the values of the post-exercise moment were different at 15 and 30 min post-exercise. In the AG, HR differed among all moments. Regarding HR, the pre-exercise value in the RG was different from those post-exercise and 15 min, while the post moment showed a difference between the 15 and 30 min values after the exercise. Finally, the 15 min moment was different from that at 30 min. Moreover, differences were found only in SBP-PEH. SBP hypotension was −10.59 ± 5.24 mmHg and −5.56 ± 7.61 mmHg in RG and AG (*p* = 0.007), respectively. DBP hypotension in AG and RG was −6.15 ± 6.41 mmHg and −6.20 ± 8.25 mmHg, respectively. No differences were found in MBP AUC between groups (GA:94.10 ± 8.42 mmHg and GF:93.95 ± 6.25 mmHg).

**FIGURE 3:**
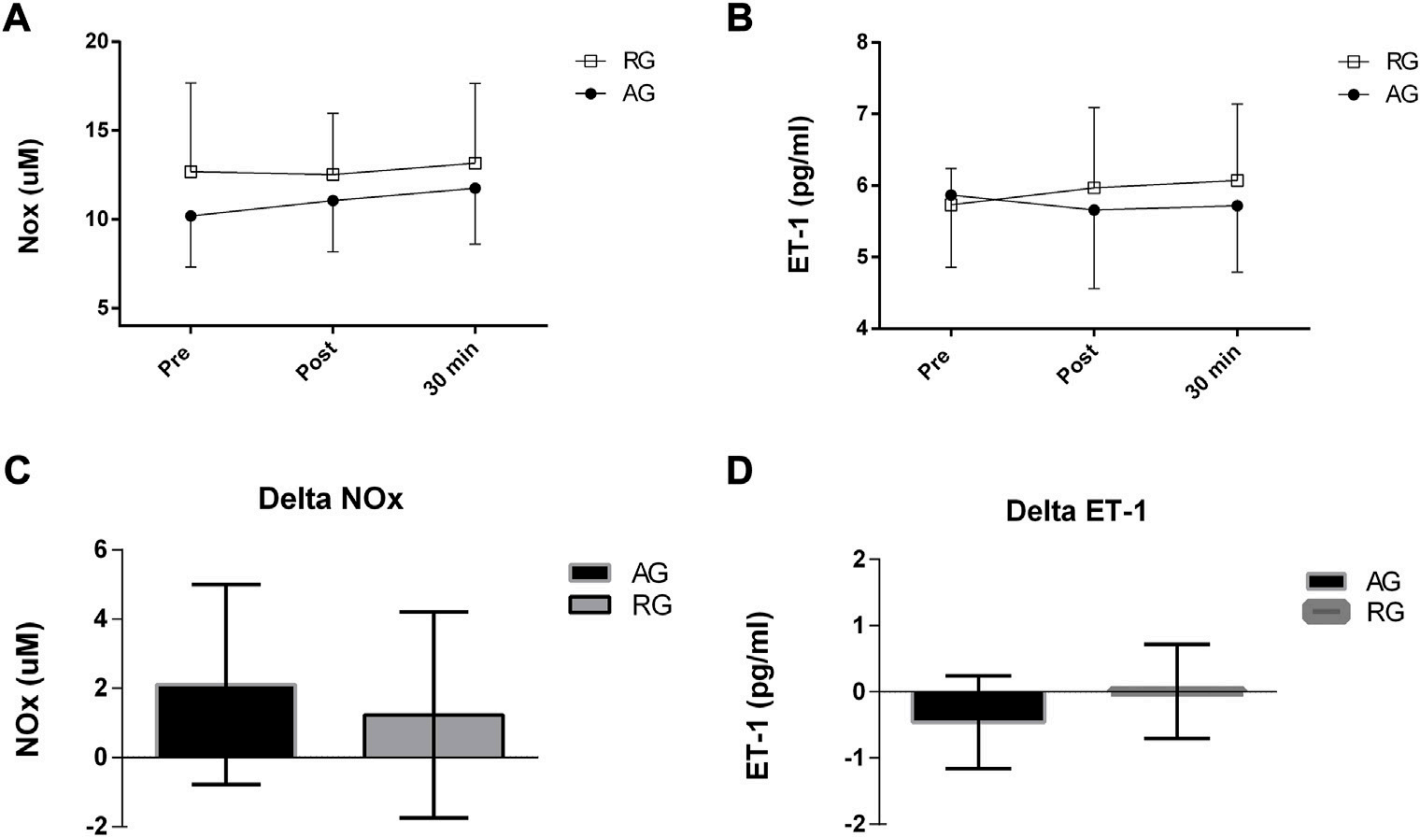
**(A-D)** Levels of endothelium-derived substances after an exercise session in middle-aged patients with hypertension. NOx, nitrites and nitrate; ET-1, endothelin-1; Delta NOx, highest post–pre values; Delta ET-1, lowest post–pre value levels. AG, aerobic group (AG); RG, dynamic resistance exercise group.

Figure 3 shows no differences in endothelium-derived substances between the groups over time.

**FIGURE 4:**
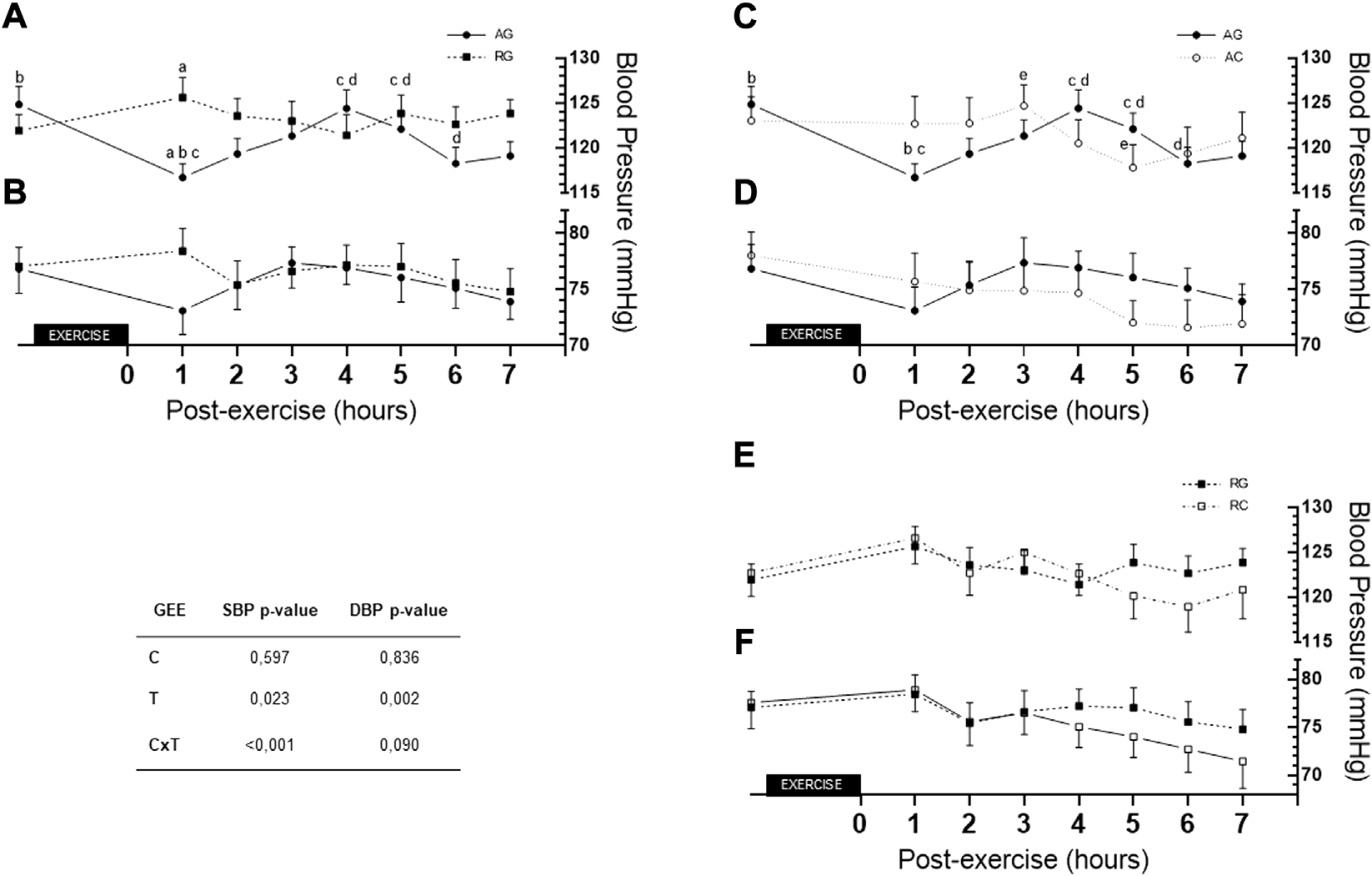
Comparison of AG and RG **(A,B)**, AG and AC **(C,D)**, and RG and RC **(E,F)** (for SBP, DBP, respectively) across the 7 h. AC—aerobic control; AG—aerobic group; C—condition factor; CxT—interaction condition x time; DBP—diastolic blood pressure; GEE—generalized estimating equation; RC—resistance control; RG—resistance group; SBP—systolic blood pressure; T—time factor. **(A)** difference between AG and RG (*p* = 0.006) in 1 h post-exercise; **(B)** difference between rest and 1 h post-exercise in AG (*p* < 0.001); **(C)** difference between 1 h with hours 4 and 5 post-exercise in AG (*p* = 0.003 and *p* = 0.027, respectively); **(D)** difference between 6 h with hours 4 and 5 post-exercise in AG (*p* = 0.022 and *p* = 0.020, respectively); **(E)** difference between hour 3 and 5 post-control session in AC (*p* = 0.016).

Figure 4 depicts differences between the AG and RG SBP at 1 h post-exercise (116.70 ± 1.51 vs. 125.6 ± 2.25, respectively, *Δ* = −8.93 ± 2.71), *p* = 0.006; AG and RC at 1 h post-exercise vs. control (116.7 ± 1.51 vs. 126.6 ± 2.86, respectively, *Δ* = −9.89 ± 3.24), *p* = 0.014. Differences were found between values at rest and 1 h post-exercise in AG (124.85 ± 1.99 vs. 116.70 ± 1.51, respectively, *Δ* = −8,15 ± 1,59; *p* < 0.001); 1 h post-exercise with 4 h post-exercise (116.70 ± 1.51 vs. 124.41 ± 2.06, respectively, *Δ* = +7.70 ± 2.0; *p* = 0.003) and 1 h post-exercise with 5 h post-exercise in AG (116.70 ± 1.51 vs. 122.11 ± 1.76, respectively, *Δ* = +5.41 ± 1.64 *p* = 0.027). Moreover, there were differences in values among that at 6 h post-exercise with those at 4 h and 5 h post-exercise (119.11 ± 1.61 vs. 124.41 ± 2.06 and 122.11 ± 1.76, *p* = 0.022 and *p* = 0.020, respectively); 3 h and 5 h post-exercise in AC (124.70 ± 2.32 vs. 117.81 ± 2.60, *p* = 0.016) under AG. Lastly, difference was found in 3 h vs. 5 h post-exercise values (124.70 ± 2.32 and 117.81 ± 2.53, respectively, *Δ* = +6.89 ± 1.99, *p* < 0.016) in AC. For DBP, there was no group or time interaction (*p* = 0.090).

**FIGURE 5:**
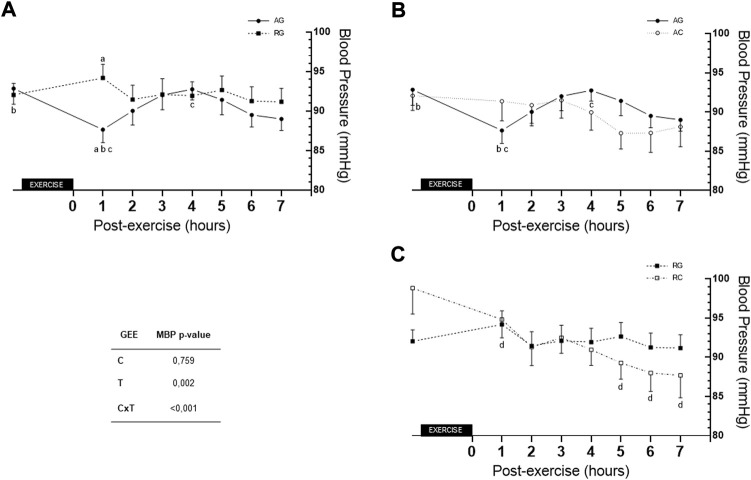
Comparison of AG and RG **(A)**, AG and AC **(B)**, and RG and RC **(C)** for MBP across the 7 h. AC—aerobic control; AG—aerobic group; c- condition factor; CxT—interaction condition x time; GEE—generalized estimating equation; MBP—mean blood pressure; RC—resistance control; RG—resistance group; T—time factor. a- difference between AG and RG (*p* = 0.006) for hour 1 post-exercise; b- difference between rest and hour 1 post-exercíse in AG (*p* = 0.001); c- difference between hour 1 and hour 4 post-exercise in AG (*p* = 0.006); d- difference between hour 1 with hours 5, 6, and 7 post-exercise in RC (*p* = 0.031, *p* = 0.002, and *p* = 0.041, respectively).

Regarding MBP over the 7 h post-exercise session, Figure 5 depicts differences between groups at 1 h. Moreover, there is difference between values at rest and 1 h post-exercise (*Δ* = −5.20 ± 1.28, *p* = 0.001) and between 1 h and 4 h post-exercise (*Δ* = −5.10 ± 1.38, *p* = 0.006). In the RC, differences were found between values at 1 h and 5 h post-exercise (*Δ* = −5.53 ± 1.69, *p* = 0.031), 6 h post-exercise (*Δ* = −6.80 ± 1.73, *p* = 0.002) and 7 h post-exercise (*Δ* = −7.12 ± 2.24, *p* = 0.041).

In addition to a pooled analysis, the subanalysis by sex of both men and women showed reduced SBP after 1 h of aerobic exercise compared with those pre-exercise (F and I, respectively). Also, women showed a reduction in SBP after 7 h compared to that in pre-exercise (K). In the grouped analysis, there was a reduction after 5 h and 6 h of aerobic exercise execution compared to that at the 4 h time point. However, this result was maintained only in women (L and M). When the type of exercise and time were fixed, men had higher SBP than women 6 h after the strength exercise (129.09 ± 2.29 and 118.19 ± 2.33, *p* = 0.001A) (Supplementary Table S1).

Unlike in the pooled analysis, in the subanalysis by sex for DBP presented in supplementary Table 2, differences were found over time mainly for the aerobic control (B) and resistance control (C, D, F, G, and H) groups. In the experimental group, women who performed strength exercises showed a reduction from hour 3 to hour 6 after the session (E) (Supplementary Table S2).

For MBP (Supplementary Table S3), women who performed aerobic exercise showed a reduction in MBP from pre- to the first hour after exercise (A). In addition, women who performed strength exercises showed a reduction after 6 h compared to 3 and 4 h after the session (B and C). There were no differences between the types of exercise in both sexes; however, the difference between men and women occurred 6 h after strength exercise (G).

Notably, SBP hypotension (up to 30 min) after aerobic exercise was negatively correlated with TXA2 (r = −0.436; *p* = 0.02). SBP and DBP before exercise showed a negative correlation with SBP hypotension after aerobic exercise (SBP: r = −0.454, *p* = 0.018; DBP: r = −0.578, *p* = 0.002). DBP hypotension after aerobic exercise was correlated with SBP and DBP pre-exercise (SBP: r = −0.446; *p* = 0.020; DBP: r = −0.570; *p* = 0.002). In contrast, SBP hypotension after strength exercise showed a negative correlation with SBP pre-exercise (SBP: r = −0.688; *p* < 0.0001) (Supplementary Table S4, S5).

After strength exercise, the average SBP at 24 h was correlated with BMI (r = 0.433; *p* = 0.024), while the DBP 24-h mean was correlated with TXA2 (r = 0.531; *p* = 0.004). The DBP of the daytime mean after aerobic exercise showed a positive correlation with VO_2peak_ (r = 0.420; *p* = 0.029). In contrast, daytime SBP was correlated with BMI (r = 0.436; *p* = 0.023) and TXA2 levels (r = 0.415; *p* = 0.031) after dynamic resistance exercise. A correlation was also found between the daytime mean DBP and TXA2 concentrations (r = 0.507; *p* = 0.007). The nighttime SBP after aerobic exercise correlated with ET-1 delta (r = 0.476; *p* = 0.012), VO_2peak_ (r = −0.432; *p* = 0.024), and BMI (r = 0.512; *p* = 0.006). The average nighttime DBP also correlated with ET-1 levels (r = 0.517; *p* = 0.006). Finally, after dynamic resistance exercise, a correlation was found between mean DBP and FMD (r = −0.410; *p* = 0.034) (Supplementary Table S4, S5).

## Discussion

This study aimed to investigate whether acute aerobic and resistance exercise sessions affect ABPM and NOx/ET-1. In addition, possible correlations between the baseline variables of middle-aged subjects with hypertension with PEH and ABPMex effects were investigated. Both groups presented with SBP-PEH, although a more pronounced hypotension was observed after aerobic exercise. Additionally, only the AG showed DBP hypotension (only clinical blood pressure). Ambulatory blood pressure did not differ between exercise (AG or RG) and control (AC and RC) sessions for 24 h. For the assessment of resting pressure for up to 7 h, there was a positive effect for the aerobic group. Similar concentrations of ET-1 and NO_x_ up to 30 min post-exercise were observed between the AG and RG.

### Office blood pressure and ambulatory blood pressure

Mean daytime HR increased after an aerobic exercise session compared to the rest in this study. Simultaneously, an increasing tendency was observed in the RG, but the difference was not statistically significant (*p* = 0.06). The literature shows that an exercise session can reduce baroreflex sensitivity, which is usually followed by an increased HR (Heffernan et al., 2007; Brito et al., 2014). After an exercise session, both groups presented a nocturnal HR decline compared to the rest. AG also promoted a nocturnal decline in the rate pressure product. A 10% decrease in blood pressure during the nighttime in relation to the daytime has an inverse relationship with cardiovascular outcomes (Ben-Dov et al., 2007). However, the nocturnal decrease in the rate pressure product was not related to the SBP. This was due to the average HR increase during the daytime after the effort, which influenced the decrease in the nighttime, as the calculation of the fall takes these variables into account. Finally, a single exercise session did not reduce the mean ambulatory blood pressure relative to the rest.

The clinical blood pressure results of the present study showed a reduction of −10.59 ± 5.24 and −5.56 ± 7.61 mmHg in SBP, post-aerobic and resistance exercise, respectively, pointing out differences between the groups for deltas. Both groups also presented a 30 min decrease in SBP. On the other hand, DBP showed a reduction of −6.15 ± 6.41 and −6.20 ± 8.25 mmHg, post-aerobic and resistance exercise, respectively; with no difference between the groups. Differences between moments were presented only in the AG (pre- and 30 min post-exercise). Moreover, a reduction of −8.15 ± 1.59 in SBP and −5.20 ± 1.28 in MBP at 1 h post-exercise was observed in the AG. Finally, at 1 h post-exercise, SBP, MBP, and AG were significantly lower than those in the RG. In addition to scientific relevance, both groups present significant results in clinical practice, as they have a potential effect on reducing cardiovascular risk (Lewington et al., 2002), especially in middle-aged individuals. For example, a 5 mmHg chronic reduction in SBP has been associated with a 9% and 14% decrease in mortality from coronary heart disease and stroke, respectively (Stamler et al., 1989; Alpsoy, 2020). This could amplify the beneficial effects of pharmacological treatment. Our results are in line with the systematic review by Rivera *et al.* (Carpio-Rivera et al., 2016), in which they stated that the post-aerobic exercise hypotension was approximately −6.22/−3.80 mmHg and after strength exercise was -3.36/-2.73 mmHg, for SBP and DBP, respectively. When all modalities were stratified for the population with hypertension, PEH showed falls of approximately −8.13/−3.02 mmHg. PEH has been widely studied in the literature because it has an important effect on the prevention and treatment of SAH. PEH seems to be of clinical importance when some studies have demonstrated a correlation between the magnitude of PEH and chronic adaptations of training on resting blood pressure (Liu et al., 2012; Hecksteden et al., 2013). Brito et al. (2018) suggested that PEH has a significant but small protective effect on the cardiovascular system despite the development of chronic adaptations and reduced cardiovascular risk with the accumulation of exercise sessions (Brito et al., 2018a). Nonetheless, in recent years, only five investigations using an exercise prescription methodology similar to that used in the present study have been found in the literature. Only one study compared the effect of continuous and interval aerobic exercise on 24 h ambulatory blood pressure in treated patients with hypertension, and both groups had reduced ambulatory blood pressure (Ciolac et al., 2009). In contrast, four studies used the moderate fatigue method to prescribe strength exercise in patients with hypertension, with all of them presenting with PEH (Queiroz et al., 2015; La Scala Teixeira et al., 2017; Queiroz et al., 2017; De Freitas Brito et al., 2019).

Individuals treated with antihypertensive drugs exhibited a negative association between pre-exercise blood pressure values with SBP and DBP post-aerobic exercise hypotension. In the RG, pre-exercise blood pressure was negatively associated only with SBP-PEH. Our results disagree with those of Carpio-Rivera et al. (2016), which showed that the PEH magnitude was independent of the initial blood pressure levels. On the other hand, in a systematic review by Casonatto and Polito (2009), the clinical status of the individuals was related to the magnitude and duration of PEH (Casonatto and Polito, 2009). Anyway, patients with either normotension or hypertension presented with PEH; however, the PEH was improved in subjects with a higher initial blood pressure level (Forjaz et al., 2000). We suggest that the main mechanism behind PEH in patients with hypertension is the decrease in cardiac output (Brito et al., 2014), whereas, in patients with normotension, the reduction in PVR would explain PEH. As a means, the cardiac output would remain high (Casonatto and Polito, 2009), and the occurrence of PEH would be less.

Additionally, other baseline variables were associated with post-exercise blood pressure outcomes. In the AG group, a higher SBP nighttime mean post-exercise was associated with a lower VO_2peak_ or a higher individual BMI. Moreover, the lower the FMD, the higher the mean DBP during the nighttime post-strength exercise. Finally, in the RG group, individuals with higher BMI presented an increase in the mean SBP 24 hours. Thus, despite the acute benefits that exercise can promote, it is perceived that physical training acts as a non-drug tool for patients with hypertension, promoting chronic benefits in maximum oxygen capacity, BMI, and endothelial function. Training helps in systemic adaptations that improve endothelial function and consequently facilitate the reduction of PVR and blood pressure after exercise (Carpio-Rivera et al., 2016). Thus, reducing and treating some risk factors through better pharmacological and non-pharmacological interventions can help in the treatment of hypertension (Braunwald, 2014).

### Vasoactive substances

Although patients with hypertension usually present with endothelial dysfunction (Brito et al., 2014), l-arginine supplementation combined with aerobic exercise can improve the diastolic hypotensive response (Neto et al., 2018). In addition, ET-1 also plays a negative role in the post-exercise vasodilator response (Boeno et al., 2019). In the present study, the concentrations of NOx and ET-1 remained unchanged until 30 min after the exercise protocols in middle-aged patients with hypertension. Some studies have reported similar results or even a decrease in endothelial function, which is explained by greater sympathetic activity and higher production of reactive oxygen species (ROS) (Goto et al., 2003; Phillips et al., 2011; Polito et al., 2011; Birk et al., 2012; Dawson et al., 2013; Franklin et al., 2014; Mcclean et al., 2015; Phillips et al., 2015). Literature indicates that the endothelium plays an important role in the control of blood pressure; however, in this context, it seems that vasodilation does not depend on NO pathways. Thus, PEH could be explained through a histamine-dependent pathway (Halliwill et al., 2013; Brito et al., 2014; Romero et al., 2017).

Decreased endothelial function is associated with a decreased release or bioavailability of vasodilatory substances due to oxidative inactivation (Valko et al., 2007). In our study, vasoactive markers were found to be associated with blood pressure. Higher baseline thromboxane levels are associated with higher SBP hypotension after aerobic exercise. Baseline thromboxane levels showed a positive association with mean DBP 24 h, mean SBP, and DBP daytime after resistance exercise. Finally, after aerobic exercise, baseline ET-1 levels were positively correlated with mean DBP. Thus, we understand that these associations can justify our results; as previously seen, a higher baseline blood pressure is related to higher PEH. Moreover, the fact that thromboxane concentrations changed the mean 24-h and daytime blood pressure may be related to the modality since this happened only in the RG group. Resistance exercise is believed to amplify the consequences of endothelial dysfunction and has repercussions on the relationship between baseline ET-1 levels and mean nighttime blood pressure. It has recently been demonstrated by our research group that high-intensity resistance exercise (80% 1RM) increases ET-1 concentrations (Boeno et al., 2019), whereas maximum voluntary contraction is related to impaired FMD (Franklin et al., 2014). However, moderate-intensity resistance exercise (50% 1RM) increases the bioavailability of NO and, consequently, the FMD (Boeno et al., 2019), which is a safe alternative for individuals with hypertension. Although these associations have rational explanations, more investigations are necessary, especially on PEH length, to the general clinical condition of the individual and not only the resting blood pressure before exercise.

### Limitations and strengths

The present study has some limitations. Subjects were under strict pharmacological treatment to lower blood pressure, which may explain our results concerning the absence of significant differences between the means of ambulatory blood pressure after exercise and that at rest. In addition, the participants did not access the FMD after the exercise session. On the other hand, individuals were in the normal blood pressure range before starting exercise sessions, as recommended. The work also contains originality since no studies with a well-controlled methodology have evaluated the responses of office blood pressure, ambulatory blood pressure, and vasoactive substances in middle-aged patients with hypertension.

## Conclusion

Aerobic and strength exercises promoted SBP-PEH, although the AG group presented with higher hypotension. Simultaneously, only aerobic exercise promoted DBP hypotension. In terms of ambulatory blood pressure, only the AG experienced SBP and MBP hypotension after exercise. Moreover, in middle-aged patients with hypertension, in the face of aerobic or resistance exercise, the NOx and ET-1 pathways were not activated; thus, it did not provide the best explanation for PEH. Finally, we found associations of characterization variables, mainly vasoconstrictive products of the endothelium, with PEH and ABPMex. We cannot confirm that these associations can affect PEH, and further studies are needed.

## Data Availability

The raw data supporting the conclusion of this article will be made available by the authors, without undue reservation.
