# Supplementary material for: Effects of exercise modalities on decreased blood pressure in patients with hypertension
Source: Front Physiol. 2022 Oct 14;13:993258. doi: 10.3389/fphys.2022.993258 (PMC9614347; doi:10.3389/fphys.2022.993258)
Supplement: Supplementary file 1 [file Table1.docx]

**Supplementary Table 1**. Comparisons within and between males and females for Systolic Blood Pressure (SBP).

|  | **MALES** | | | | | | **FEMALES** | | | | | |  |
| --- | --- | --- | --- | --- | --- | --- | --- | --- | --- | --- | --- | --- | --- |
| **Time / Group** | **AG** | **AC** | **RG** | **RC** | **p**  **(time)** | **p**  **(group)** | **AG** | **AC** | **RG** | **RC** | **p**  **(time)** | **p**  **(group)** | **p**  **(GxTxS)** |
| **Pre exercise** | 124.08 (2.29)^F^ | 119.33 (2.44) | 125.18 (2.70) | 125.63 (4.13) | 0.011^F^ |  | 125.57 (3.18)^J, K^ | 125.92 (3.82) | 119.69 (2.24) | 125.25 (2.10) | 0.006^J^  0.017^K^ |  |  |
| **1h post** | 116.08 (1.67)^E, F^ | 120.23 (3.00) | 123.09 (2.60) | 123.91 (3.81) | 0.033^E^ |  | 117.29 (2.46)^J^ | 125.00 (5.07) | 127.37 (3.28) | 128.44 (4.00)^O^ | 0.003^O^ |  |  |
| **2h post** | 119.31 (2.78) | 121.08 (2.96) | 121.18 (3.40) | 121.18 (3.44) |  |  | 119.36 (2.18) | 124.29 (4.75) | 125.19 (2.28)^N^ | 123.75 (3.49) | 0.013^N^ |  |  |
| **3h post** | 121.62 (2.15) | 125.08 (2.13)^H^ | 123.18 (3.24)^G^ | 127.80 (2.15)^I^ | 0.039^G^  0.021^H^  0.013^I^ |  | 121.07 (2.73) | 124.36 (4.03) | 122.88 (2.92) | 123.25 (3.74) |  |  |  |
| **4h post** | 122.85 (2.07)^E^ | 120.54 (2.80) | 125.64 (3.54) | 124.56 (2.12) |  |  | 125.86 (3.43)^L^ | 120.50 (4.28) | 118.50 (2.79) | 121.50 (3.57) | 0.031^L^ |  |  |
| **5h post** | 121.54 (1.83) | 117.92 (2.39)^B, H^ | 127.18 (2.48)^B^ | 120.30 (0.96)^I^ |  | 0.043^B^ | 122.64 (2.94)^M^ | 117.71 (4.35) | 121.56 (2.82) | 120.00 (4.05) | 0.040^M^ |  |  |
| **6h post** | 119.92 (2.30)^C^ | 118.00 (3.47)^D^ | 129.09 (2.29)  ^A, C, D, G^ | 123.20 (3.08) |  | 0.028^C^  0.046^D^ | 116.71 (2.68)  ^L, M^ | 120.77 (4.70) | 118.19 (2.33)^A, N^ | 116.25 (4.04)^O^ |  |  | 0.001^A^ |
| **7h post** | 120.08 (2.40) | 122.23 (2.72) | 126.36 (2.47) | 124.40 (3.36) |  |  | 118.21 (2.14)^K^ | 120.00 (5.05) | 122.13 (1.87) | 118.56 (4.73) |  |  |  |

AC: aerobic control; AG: aerobic group; p (GxTxS): comparison between sexes fixing type of exercise and time; RC: resistance control, RG: resistance group; p (group): comparison between types of exercise in same time; p (time): comparison across the time to same type of exercise.
