# Supplementary material for: Effects of exercise modalities on decreased blood pressure in patients with hypertension
Source: Front Physiol. 2022 Oct 14;13:993258. doi: 10.3389/fphys.2022.993258 (PMC9614347; doi:10.3389/fphys.2022.993258)
Supplement: Supplementary file 2 [file Table2.docx]

**Supplementary Table 2**. Comparisons within and between males and females for Diastolic Blood Pressure (DBP).

|  | **MALES** | | | | | | **FEMALES** | | | | | |  |
| --- | --- | --- | --- | --- | --- | --- | --- | --- | --- | --- | --- | --- | --- |
| **Time / Group** | **AG** | **AC** | **RG** | **RC** | **p**  **(time)** | **p**  **(group)** | **AG** | **AC** | **RG** | **RC** | **p**  **(time)** | **p**  **(group)** | **GxTxS** |
| **Pre exercise** | 77.38  (2.20) | 80.50  (2.48) | 77.64  (2.07) | 82.00  (2.83)^C, D^ | 0.032^C^  0.005^D^ |  | 76.36  (3.63) | 75.77  (3.26) | 76.69  (2.44) | 79.77  (1.91) |  |  |  |
| **1h post** | 74.69 (1.97) | 76.15  (2.50) | 78.45  (2.58) | 77.64  (3.29) |  |  | 71.64  (3.57) | 75.29  (4.32) | 78.44  (2.88) | 79.75  (3.02)^F, G^ | <0.001^F^  0.011^G^ |  |  |
| **2h post** | 75.38  (2.87) | 75.77  (2.91) | 72.36  (2.80) | 70.55  (3.16)^C^ |  |  | 75.36  (3.21) | 74.14  (3.94) | 77.50  (2.93) | 79.06  (3.33) |  |  |  |
| **3h post** | 78.77  (2.74) | 76.85  (2.19)^B^ | 72.36  (2.51) | 74.70  (2.68) | 0.024^B^ |  | 76.07  (3.40) | 73.07  (4.25) | 79.56  (3.03)^E^ | 77.06  (3.27) | 0.021^E^ |  |  |
| **4h post** | 78.23  (1.45)^A^ | 75.69  (2.14) | 78.09  (3.30) | 71.78  (1.96)^A^ |  | 0.049^A^ | 75.71  (2.45) | 73.71  (4.13) | 76.56  (1.93) | 77.31  (3.18)^H^ | 0.013^H^ |  |  |
| **5h post** | 78.08  (1.78) | 72.31  (2.81) | 79.45  (3.54) | 73.30  (3.26) |  |  | 74.21  (3.80) | 71.79  (2.77) | 75.38  (2.38) | 74.75  (2.87) |  |  |  |
| **6h post** | 76.54  (2.22) | 71.85  (3.13) | 78.64  (3.36) | 75.90  (3.60) |  |  | 73.79  (2.71) | 71.77  (3.91) | 73.44  (2.54)^E^ | 71.25  (3.06)^F, H^ |  |  |  |
| **7h post** | 74.77  (2.27) | 71.69  (2.54)^B^ | 79.18  (3.27) | 71.90  (3.59)^D^ |  |  | 73.14  (2.15) | 72.38  (4.68) | 71.81  (2.34) | 71.38  (4.07)^G^ |  |  |  |

AC: aerobic control; AG: aerobic group; p (GxTxS): comparison between sexes fixing type of exercise and time; RC: resistance control, RG: resistance group; p (group): comparison between types of exercise in same time; p (time): comparison across the time to same type of exercise.
