# Supplementary material for: Effects of exercise modalities on decreased blood pressure in patients with hypertension
Source: Front Physiol. 2022 Oct 14;13:993258. doi: 10.3389/fphys.2022.993258 (PMC9614347; doi:10.3389/fphys.2022.993258)
Supplement: Supplementary file 3 [file Table5.docx]

**Supplementary table 5:** Correlations between post-exercise hypotension or ambulatory blood pressure monitoring after dynamic resistance session with the other variables.

| Variables | SBP hypotension | 24-h SBP | 24-h DBP | Daytime SBP | Daytime DBP |
| --- | --- | --- | --- | --- | --- |
| SBP before exercise | r=-0.688; P<0.0001 | _ | _ | _ | _ |
| TXA2 | _ | _ | r=0.531; p=0.004 | =0.415; p=0.031 | r=0.507; p=0.007 |
| BMI | _ | r=0.433; p=0.024 | _ | r=0.436; p=0.023 | _ |
| FMD | _ | _ | r=-0.410; p=0.034 | _ | _ |
|  |  |  |  |  |  |

SBP, systolic blood pressure; DBP, diastolic blood pressure; TXA2, Thromboxanes; BMI: body mass index; FMD, flow-mediated dilation; **the statistical differences (p<0.05).**
