# Supplementary material for: Effects of exercise modalities on decreased blood pressure in patients with hypertension
Source: Front Physiol. 2022 Oct 14;13:993258. doi: 10.3389/fphys.2022.993258 (PMC9614347; doi:10.3389/fphys.2022.993258)
Supplement: Supplementary file 4 [file Table3.docx]

**Supplementary Table 3**. Comparisons within and between males and females for Mean Blood Pressure (MBP).

|  | **MALES** | | | | | | **FEMALES** | | | | | |  |
| --- | --- | --- | --- | --- | --- | --- | --- | --- | --- | --- | --- | --- | --- |
| **Time / Group** | **AG** | **AC** | **RG** | **RC** | **p**  **(time)** | **p**  **(group)** | **AG** | **AC** | **RG** | **RC** | **p**  **(time)** | **p**  **(group)** | **GxTxS** |
| **Pre exercise** | 92.94  (2.00) | 93.43  (2.07) | 93.49  (1.77) | 95.38  (2.55) |  |  | 92.76  (3.34)^A^ | 92.49  (3.12) | 91.02  (2.12) | 95.13  (1.86) | 0.001^A^ |  |  |
| **1h post** | 88.48  (1.44) | 90.86  (2.37) | 93.34  (2.18) | 93.08  (3.23) |  |  | 86.85  (2.85)^A^ | 91.85  (4.25) | 94.74  (2.53) | 95.99  (3.18)^D, E^ | <0.001^D^  0.013^E^ |  |  |
| **2h post** | 90.02  (2.37) | 90.88  (2.33) | 88.62  (2.41) | 87.42  (2.96) |  |  | 90.02  (2.67) | 90.86  (3.90) | 93.38  (2.48)^B^ | 93.96  (3.28) | 0.004^B^ |  |  |
| **3h post** | 93.03  (2.18) | 92.92  (1.87) | 89.32  (2.53) | 92.39  (2.16) |  |  | 91.07  (2.91) | 90.17  (3.98) | 94.01  (2.76)^C^ | 92.46  (3.07) | 0.013^C^ |  |  |
| **4h post** | 93.10  (1.25) | 90.63  (2.07) | 93.94  (3.19) | 89.35  (1.35) |  |  | 92.42  (2.35) | 89.31  (3.90) | 90.54  (1.92) | 92.04  (3.17)^F^ | 0.021^F^ |  |  |
| **5h post** | 92.56  (1.61) | 87.51  (2.44) | 95.36  (2.88) | 88.97  (2.31) |  |  | 90.36  (3.30) | 87.09  (3.19) | 90.77  (2.18) | 89.83  (3.09) |  |  |  |
| **6h post** | 91.01  (1.70) | 87.23  (3.12) | 95.45  (2.55)^G^ | 91.66  (3.16) |  |  | 88.11  (2.38) | 88.11  (3.97) | 88.36  (2.26)  ^B, C, G^ | 86.25  (3.26)^D, F^ |  |  | 0.038^G^ |
| **7h post** | 89.87  (2.03) | 88.55  (2.38) | 94.92  (2.78) | 89.39  (3.35) |  |  | 88.17  (2.02) | 88.26  (4.60) | 88.58  (1.88) | 87.09  (4.14)^E^ |  |  |  |

AC: aerobic control; AG: aerobic group; p (GxTxS): comparison between sexes fixing type of exercise and time; RC: resistance control, RG: resistance group; p (group): comparison between types of exercise in same time; p (time): comparison across the time to same type of exercise.
