# Supplementary material for: Effects of exercise modalities on decreased blood pressure in patients with hypertension
Source: Front Physiol. 2022 Oct 14;13:993258. doi: 10.3389/fphys.2022.993258 (PMC9614347; doi:10.3389/fphys.2022.993258)
Supplement: Supplementary file 5 [file Table4.docx]

**Supplementary table 4:** Correlations between post-exercise hypotension or ambulatory blood pressure monitoring after aerobic session with the other variables.

| Variables | SBP hypotension | DBP hypotension | Daytime DBP | Night-time SBP | Night-time DBP |
| --- | --- | --- | --- | --- | --- |
| SBP before exercise | r=-0.454; p=0.018 | r=-0.446; p=0.020 | _ | _ | _ |
| DBP before exercise | r=-0.578; p=0.002 | r=-0.570; p=0.002 | _ | _ | _ |
| TXA2 | r= -0.436; p=0.02) | _ | _ | _ | _ |
| VO_2peak_ | _ | _ | r=0.420; p=0.029 | r=-0.432; p=0.024 | _ |
| ET-1 delta | _ | _ | _ | r=0.476; p=0.012 | r=0.517; p=0.006 |
| BMI | _ | _ | _ | r=0.512; p=0.006 | _ |

SBP, systolic blood pressure; DBP, diastolic blood pressure; TXA2, Thromboxanes; VO_2_, Peak oxygen consumption; ET-1: endothelin-1; BMI: body mass index; **the statistical differences (p<0.05).**
